# The Red Queen Race between Parasitic Chytrids and Their Host, *Planktothrix*: A Test Using a Time Series Reconstructed from Sediment DNA

**DOI:** 10.1371/journal.pone.0118738

**Published:** 2015-03-20

**Authors:** Marcia Kyle, Sigrid Haande, Veronika Ostermaier, Thomas Rohrlack

**Affiliations:** 1 Norwegian University of Life Sciences (NMBU), Environmental Sciences, Ås, Norway; 2 Norwegian Institute for Water Research (NIVA), Oslo, Norway; University of Shiga Prefecture, JAPAN

## Abstract

Parasitic chytrid fungi (phylum *Chytridiomycota)* are known to infect specific phytoplankton, including the filamentous cyanobacterium *Planktothrix*. Subspecies, or chemotypes of *Planktothrix* can be identified by the presence of characteristic oligopeptides. Some of these oligopeptides can be associated with important health concerns due to their potential for toxin production. However, the relationship between chytrid parasite and *Planktothrix* host is not clearly understood and more research is needed. To test the parasite - host relationship over time, we used a sediment core extracted from a Norwegian lake known to contain both multiple *Planktothrix* chemotype hosts and their parasitic chytrid. Sediment DNA of chytrids and *Planktothrix* was amplified and a 35-year coexistence was found. It is important to understand how these two antagonistic species can coexistence in a lake. Reconstruction of the time series showed that between 1979–1990 at least 2 strains of *Planktothrix* were present and parasitic pressure exerted by chytrids was low. After this period one chemotype became dominant and yet showed continued low susceptibility to chytrid parasitism. Either environmental conditions or intrinsic characteristics of *Planktothrix *could have been responsible for this continued dominance. One possible explanation could be found in the shift of *Planktothrix* to the metalimnion, an environment that typically consists of low light and decreased temperatures. *Planktothrix* are capable of growth under these conditions while the chytrid parasites are constrained. Another potential explanation could be due to the differences between cellular oligopeptide variations found between *Planktothrix* chemotypes. These oligopeptides can function as defense systems against chytrids. Our findings suggest that chytrid driven diversity was not maintained over time, but that the combination of environmental constraints and multiple oligopeptide production to combat chytrids could have allowed one *Planktothrix* chemotype to have dominance despite chytrid presence.

## Introduction

Chytrid fungi (phylum Chytridiomycota) have been found to be common pelagic phytoplankton parasites [1, 2]. In this role, they often occupy a significant position in aquatic food webs, first by parasitizing and removing particularly susceptible phytoplankton species, allowing for increased diversity, and secondly by being a rich source of nutrients for zooplankton [3,4]. Due to their typically narrow host range, chytrids are able to exert significant selective pressures on specific strains and in turn on phytoplankton populations [5].

However, regardless of the potential for high growth rates, there can be limitations on chytrid proliferation. An important stage in the chytrid life cycle is the free-swimming zoospore whose task is to find the correct host before the zoospore’s limited internal nutrient stores are depleted [6]. Gerphagnon et al. [6] utilized another filamentous cyanobacteria, *Anabaena macrospora*, to identify the stages of infection. Once attached, the zoospore injects its contents into the cell and a prosporangium is formed. A rhizoid is sent from the prosporangium through the cell, releasing enzymes, such as serine proteases used for cellular digestion of the host in order to obtain nutrients from the host. An epiphytic bud, the sporangium, is subsequently formed for the production of zoospores. Upon maturation these zoospores are released to begin the cycle again. This cycle of infection by chytrids results in rapid host filament fragmentation, cell rupture and death [6, 1]. However, if host density is low, zoospores are less likely to find the specific host phytoplankton before internal nutrient stores are exhausted. Likewise, environmental constraints can also apply pressure to the chytrids. For example both cold temperatures and low light have been shown to have negative effects on chytrid fitness [2, 4, 8]. These limitations and stresses for the chytrids can be used as an advantage by the phytoplankton hosts.

De Bruin et al. [7] investigated the parasite—host relationship using chytrids and the diatom *Asterionella formosa* as models. They applied the “Red Queen” theory [9] to describe the co-evolutionary arms race where host adaption skills require continual improvement to survive the parasitism pressure. Failure of either parasite or host to adapt to each new challenge can lead to a loss of fitness resulting in a decrease in the frequency of the genotype in the next generation. In laboratory studies, serial passage techniques were used to observe the fitness response of chytrid parasites presented with either single or multi-strain cultures of *A*. *formosa* over time [7]. Their results showed that after 200 generations, chytrid fitness, described in this study as the difference between primary and secondary infections, was increased when presented with single diatom strains. In fact, this pattern began to form as early as 100 generations. On the contrary, when chytrids were presented with multi-strain diverse diatom cultures, chytrid fitness did not increase. They concluded that when host genetic diversity was high, chytrids were unable to adapt to this diversity enough to increase fitness. However, chytrids were able to adapt rapidly to monoculture hosts. The results showed that chytrid parasitism could be the driving force in diversification of a host population in accordance with the “Red Queen Theory”.

Chytrids also have been found to parasitize the filamentous cyanobacterium *Planktothrix*, and can rapidly decimate *Planktothrix* bloom events [1]. Within *Planktothrix* significant differences can be found between the types and numbers of oligopeptides present within the cell. It is with these oligopeptide differences that subpopulation chemotypes are determined within *Planktothrix* [10]. The oligopeptides found within the chemotypes are highly varied and can, for example, act as inhibitors of the serine proteases released by the chytrid rhizoids during cell invasion. Although these oligopeptides may have many as yet unknown functions, studies have shown that they can act as defensive mechanisms against the internal invasion typical of chytrid parasitism [11].

Chytrids known to specifically parasitize *Planktothrix* have been identified in Kolbotnvannet (59°48' 7.84"N and 10°48' 8.51"E) an interesting and well-studied lake in southern Norway. The lake has been subjected to increasing stress due to rapid urbanization. Monitoring by the Norwegian Institute of Water Research (NIVA). began in Kolbotnvannet around 1970 and increased in frequency and intensity during the 1980s when nutrient loads of nitrogen (N) and phosphorus (P) were very high and the lake was highly eutrophic. Monitoring continues to the present. Remediation efforts have been varied and ongoing since the 1970s, resulting in the steady and substantial decrease in nutrient inputs and a change in the lake’s current classification from eutrophic to mesotrophic. Lake recovery however has not been uneventful. For instance, in 2005 a pipe leaked sewage into the lake for an unknown period of time before discovery, increasing the nutrient levels briefly. Regardless of remediation efforts, *Planktothrix* populations noted in 1980 remain present to date, and are of great public concern. Recreational restrictions have periodically been enforced due to the high level of toxins in the lake associated with *Planktothrix*.

In addition to lake monitoring, clonal *Planktothrix* cultures were established by NIVA between 1964 and 2008 from strains isolated from Kolbotnvannet and other surrounding lakes. Isolation of the cultures had a success rate of more than 50% [10]. These cultures are presently maintained by NIVA in their Culture Collection of Algae (NIVA-CCA; https://niva-cca.no). The *oci*B gene cluster associated with oligopeptides has been sequenced for all *Planktothrix* cultures and the resulting phylogenetic data correlated with the chemotype subpopulation classification [12]. The resulting phylogeny indicates that Kolbotnvannet has had two primary chemotypes, chemotype 1 and chemotype 9. Typically there are four distinct chemotypes found to dominate most lakes in southeastern Norway. However the other two chemotypes, 5 and 7, were not found among the filaments isolated from Kolbotnvannet. It is possible that this random sampling might have missed these common chemotypes. The phylogenetic data did indicate three minor variants that were closely related to chemotype 9. Chytrids have been found actively parasitizing *Planktothrix* in this lake and have been isolated by NIVA. These cultures are currently maintained at the Norwegian University of Life Sciences (NMBU) in the Environmental Sciences Section in Ås, Norway. Early testing the chytrids using a wide range of cyanobacteria cultures as hosts has revealed that these chytrids have a narrow and very specific host range for *Planktothrix* [1].

In the present field study, we compare chytrid infection of the two *Planktothrix* chemotypes from Kolbotnvannet to test De Bruin’s hypothesis of chytrid driven diversification of phytoplankton. To study this interaction over time, we use a dated sediment core as a biological archive and extracted DNA to reconstruct a time-course. We tested the hypothesis that chytrids drive *Planktothrix* diversity, as was shown between chytrid and diatoms. This would mean that the presence of multiple *Planktothrix* chemotypes would be correlated with stable chytrid fitness, expressed by low variation in the ratios of chytrid DNA / *Planktothrix* DNA. Likewise, during periods of high single chemotype DNA concentrations, chytrid fitness would increase, as shown by an increase in chytrid DNA, followed by a marked decrease in single chemotype DNA before subsequently returning to the multiple chemotype state. Using sediment DNA to test these ecological questions allowed us the unique opportunity to study the temporal development of parasite—host interactions in a natural field experiment.

## Materials and Methods

### Sediment core processing

The lake Kolbotnvannet (59°48' 7.84"N and 10°48' 8.51"E), located in southeastern Norway, has experienced increased urbanization during the past 35 years. NIVA has been contracted to monitor Kolbotnvannet from the early 1970s to present [13] at the request of the local community, Oppegård kommune, which has responsibility for management of this lake. This lake has no endangered species and the local municipality does not require permits for NIVA activities other than notification of sampling dates prior to lake access. Typically, monitoring has been performed monthly during the ice-free period between May and September, with the exception of the years between 1990 and 2000 when the monitoring was every second summer. Monitoring data for our study has been collected from the top four meters of the lake during the ice-free sampling periods, between 1979 and 2013, and averaged by year.

Kolbotnvannet sediment was cored on 28 June 2013 at the deepest part of the lake. Two cores (50cm each) were taken using a gravity corer at a depth of 17m. The cores were returned to the lab and maintained in the dark at ~10°C for less than 24 hours before processing. Cores were sliced in 1cm sections, starting with the sediment surface of the core. Material from one core was used for dating measurements while the other core was used to analyze wet to dry determination (water content), loss on ignition (LOI), and for DNA extraction.

Water content and dry weight (DW) were determined by drying pre-weighed samples at 60°C and reweighing. The LOI was obtained by combustion of the dry sample at 500°C for two hours followed by reweighing, and calculations were made to determine the percent of organic content (OM) of each core layer. Dating and sedimentation rates were based on determination of the sediment core layer with the highest ^137^Cs concentration and this depth was used as a marker for 1986, the date of the Chernobyl accident fallout across Norway. To analyze ^137^Cs content samples were dried at 60°C in scintillation vials then ^137^Cs was measured on the dry sediment at the Norwegian University of Life Sciences (NMBU) Isotope Laboratory using a Sodium-Iodine detector (Wallac 1480 Wizard 3" gamma counter, PerkinElmer), based on the ^137^Cs peak at 662 keV. Core depth was converted to age by assuming that the average sediment accumulation rate since 1986 also applied before 1986.

### Molecular analysis

Sediment DNA was extracted within 5 days of coring using PowerSoil DNA Isolation kit (MoBio Laboratories, Inc., Carlsbad, CA USA; cat.no. 12888) as per manufacturer’s instruction with the exception that the samples were processed without initial centrifugation to remove the aqueous portion of sediment sample. Instead, sample concentrations were corrected following quantitative real-time PCR (qPCR) to represent the amount of DNA amplified from only the OM fraction by using the parallel DW and LOI data sets.

*Planktothrix* and chytrid standards were created using two different methods. *Planktothrix* DNA, for used as standards, was isolated individually from the following cultures, with chemotype affiliations in parenthesis: NIVA-CYA 98 (chemotype 1), NIVA-CYA 407 (chemotype 5), NIVA-CYA 56/3 (chemotype 7), and NIVA-CYA 405 (chemotype 9). All strains were grown in batch culture at 20^°^C and 3–5 μmol m^−2^ s^−1^ light using Z8 as the medium, and harvested in the exponential growth phase. Filaments were centrifuged and the pellets transferred to the initial tube of the extraction kit and processed using the same method as the sediment samples. Following extraction, NanoDrop (Thermo Scientific, USA) spectrophotometric quantification was used to determine total genomic DNA concentration of each *Planktothrix* chemotype.

The chytrid standard was developed from a chytrid culture, NIVA-Chy-Lys 2009, originally isolated by NIVA from Lyseren, a lake in the same region of Norway as Kolbotnvannet, and cultured using *Planktothrix* as a host (NIVA-CYA630, classified as chemotype 7). These chytrid cultures are now maintained and available at NMBU rather than at NIVA. Environmental conditions were the same as for the *Planktothrix* cultures. The chytrids were collected by filtering the culture through 10μm-pore mesh to separate the *Planktothrix* host from the fungi zoospores and then further separated from the medium by centrifugation after which the pellet of zoospores was removed by pipette for extraction. Because of the potential for contamination of the chytrid standards with *Planktothrix* cells, cloning was used to develop the chytrid standards. PCR was performed directly from the chytrid pellet using the primers developed for chytrid quantification in sediment samples (see primer design given below and Table 1). The amplification product was then cloned according to standard procedures and sequenced using vector primers. The plasmids were linearized and quantified by NanoDrop Spectrophotometric measurements prior to use. Therefore, the differences in standard development resulted in *Planktothrix* standards based on extracted total DNA of the filaments and chytrid standards based on a short fragment of the DNA in a vector. Standard DNA concentrations were all determined similarly using NanoDrop for measurements.

**Table 1:** PCR conditions.

Pimer	bp	NIVA-CYA	Forward(5’ to 3’)	Reverse (5’ to 3’)	Probe(5’ to 3’)	ext (°C)	F
t1	133	98	TAGTTGCCTACGTTATCCCC	AAAATGACAAAGGCACTAGGAAC	TGCTTGGTGTTAATGAACTGCG	58	HEX
Cht5	135	407	GCCATGAAGCCTTGAGGACTAAT	GAGAGGCAATTTCCTGTTCACG	TGGACAGTGACCATCGTTGATTTACAACCT	61	FAM
Cht7	157	56/3	CCAAACAGAGGGGATTTCTACT	CGATCCACTTTACCATTAGACG	AGTTTGCTTGCTGTTACGGAACTG	58	FAM
Cht9	137	405	GCTAATTATTCTCCCCTTCCTCA	GCCTGAGTATTACTAATAGGTTGC	TCGTAGAGAGTCGTCACAACCG	61	FAM
chytrid	90	Chy-Lys 2009	CGTAATGTGAATTGCAGAATTCCG	ACATTAGATTCTCAAACAGGCATACC	NA	60	SYB

PCR Primer sequences for *Planktothri*x chemotypes (Cht) 1, 5, 7, 9, and chytrids with Norwegian Institute for Water Research Culture Collection of Algae, NIVA-CCA, culture identifications, *Planktothrix* probes, and also including DNA base pair length, extension (ext), temperatures, and fluorochrome (F).

Primers and probes for the quantification of *Planktothrix* chemotypes were defined in the *Planktothrix* cyanopeptoline gene cluster *oci*B region. Four *Planktothrix* genotypes that corresponded to the classification of chemotypes based on the differential expression of oligopeptides in strains by Rohrlack et al. [10] were distinguished by polymorphisms within the *oci*B gene fragment. Based on work by Sogge et al. [12] a sequence alignment of strains with known chemotype affiliation was made using BioEdit (version 7.2.0) and uniform regions were identified for each of the four genotypes. Regions of identity between genotypes and maximum sequence variability to the other genotypes were chosen.

Chytrid primers were developed based on sequencing of two *Planktothrix* associated chytrid strains published by Sønstebø and Rohrlack [1]. Utilizing the sequences of the cultures Chy-Lys2009 and Chy-Kol2008 from the NIVA Culture Collection of Algae (NIVA-CCA), an identical region was identified between these strains in the ITS gene region and a primer set, Chytrid-All, was defined that would amplify both strains.

Amplification of *Planktothrix* DNA was performed in duplicate using Fast qPCR MasterMix Plus No ROX dTTP (Eurogentec product number RT-QP2X-03+NRWOUNF) with a BioRad CFX96 Real-Time PCR Detection System (Bio-Rad, USA). The qPCR thermal cycling parameters were first optimized for all primers and probes before measuring the environmental samples. The initial denaturation of 95^°^C for 5 minutes was set, followed by 50 cycles of a two-step protocol, including denaturation at 95^°^C for 15 seconds and an annealing/extension step for 30 seconds. Optimization resulted in different annealing temperatures depending on the target (Table 1). The total PCR reaction volume was 25 μL, including 300 nM of each primer, 100nM probe, 5 μL of template DNA, and 12.5 μL of 2X Master mix per well. Bovine serum albumin (BSA, VWR #786006) was added with a final concentration of 0.2μg μL^−1^ to limit potential inhibition due to the sediment matrix. After initial testing, sediment DNA was diluted 1:50 with nuclease free water prior to amplification to avoid possible inhibition.

For the detection of chytrids, DNA was amplified using Ssofast EvaGreen kit (BioRad catalog number 172–5200). A two-step protocol was optimized for BioRad CFX96^TM^ with the initial denaturation of 98^°^C for 2 minutes followed by 50 cycles of denaturation at 98°C for 4 seconds and annealing at 60^°^C for 4 seconds. A melt curve analysis was performed on all standards and samples following amplification, and consisted of a temperature range increase from 65 to 95°C at 0.2°C increments to assess GC content. The resulting melt curves were then used to exclude amplified sediment DNA showing melt curves outside those of the chytrid standard melt curves. Total PCR reaction volume for chytrid analysis was 25 μL per well, including 300 nM of each primer, 5μL of template DNA, 2.5 μL of BSA (final concentration 0.2 μg μL^−1^), nuclease free water, and 12.5 μL of EvaGreen supermix 2X Master mix.

All *Planktothrix* and chytrid primers and probes were tested to ensure specificity. BLAST (Basic Local Alignment Search Tool developed by The National Center for Biotechnology Information) analysis was performed using the four *Planktothrix* chemotypes primers and probes and the general Chytrid-All primers. In addition, chemotypes 1, 5, 7, and 9, and Chytrid-All primers were cross-tested using all combinations of primer sets and standards to ensure specificity between the chemotypes and chytrids was possible. The Chytrid-All primer set, which was designed to amplify a shared sequence area between Chy-Lys2009 and Chy-Kol2008 was tested to ensure amplification of DNA from all chytrid cultures found at that time in the NIVA-CCA collection. The cultures were isolated from four local lakes and grown using four different *Planktothrix* strains as hosts (Chy-Stein2010/CYA-407, Chy-Lys2009/CYA-630, Chy-Kol2008/CYA-98, and Chy-Hål2010/CYA-521). These chytrid cultures now reside and are available at the NMBU’s Environmental Sciences Department, in Ås, Norway.

Phylogenetic analysis by Sogge et al. [12] found an additional three strains isolated from Kolbotnvannet that were similar to chemotype 9. The three variants were NIVA-CYA 15, 597, and 604 in the culture collection. Based on the research findings these cultures were also tested using the four *Planktothrix* primer sets.

Sediment DNA was analyzed in triplicate for each of the four *Planktothrix* chemotype primer sets (chemotype 1, 5, 7 and 9) and the Chytrid-All primers (Table 1). Abundances were determined by using the appropriate chemotype or chytrid standard curve to estimate concentrations of sample unknowns and these results were averaged for each centimeter of the core. Any amplification beyond 40 cycles was treated as a non-detect. A square root correction was applied to all data in order to handle the non-detect DNA signals. Data was normalized to the 1979 relationship between chytrids and chemotypes and between chemotypes alone.

In addition, using the results from a study by Rohrlack et al. [10] a list of all oligopeptides found in the four common *Planktothrix* was compiled.

Graphical presentations were performed using R statistical computing, version 2.15.2 [14].

## Results

### Lake monitoring

**Fig 1:**
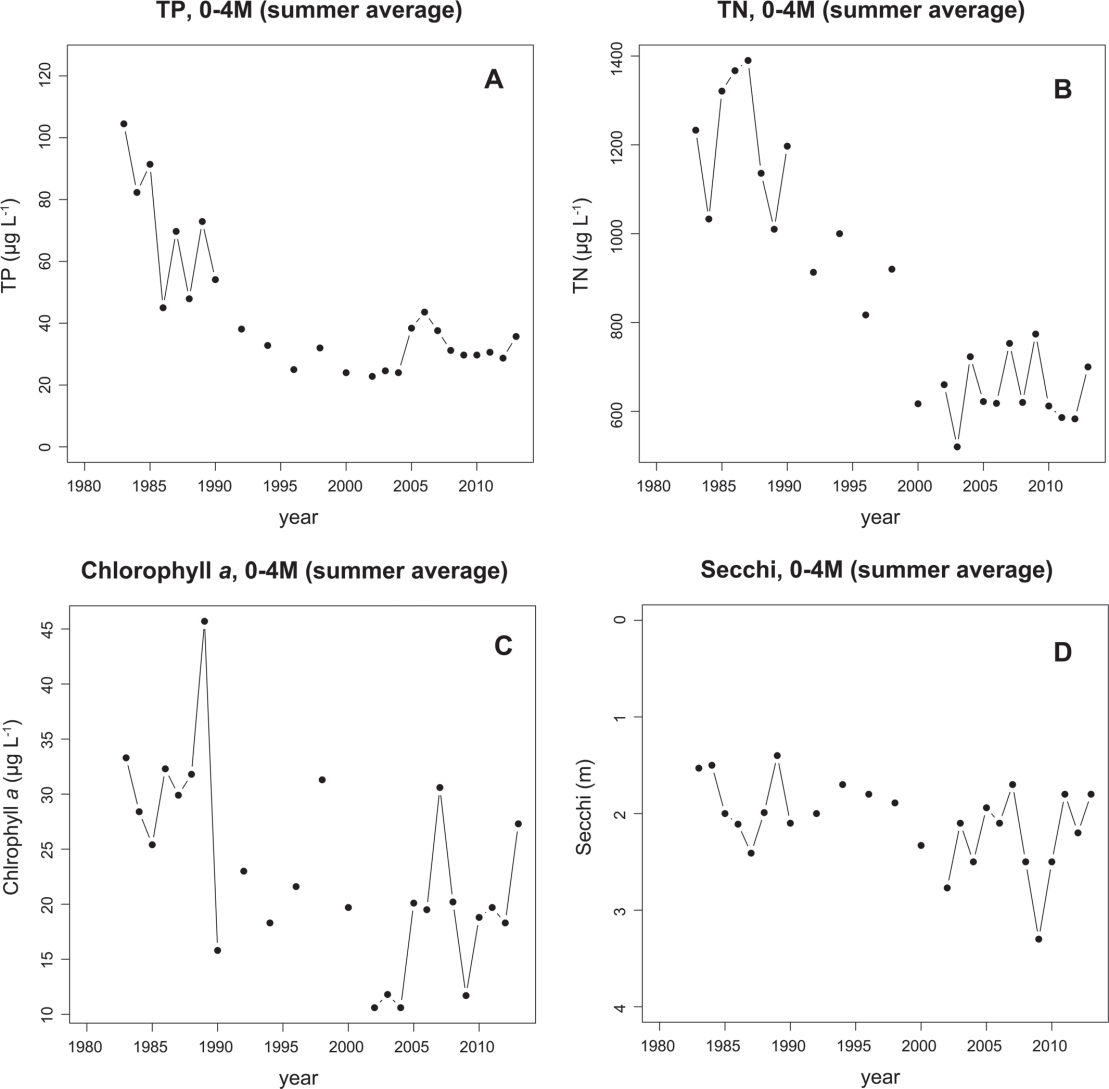
Summary of Kolbotnvannet monitoring data. Monitoring data for total phosphorus (TP), total nitrogen (TN), chlorophyll *a* (Chl.*a*) and Secchi depth for lake Kolbotnvannet in southern Norway from 1980 to 2013. Data were collected by the Norwegian Institute for Water Research, NIVA, yearly (line connecting solid circle) or every two years (solid circles without lines) and represent means over the summer months, May to September covering 0 to 4 meters depth.

Monitoring data (Fig. 1) was compiled by averaging annually the measurements taken from the top four meters during the ice-free period (May to August) since 1980 [13]. Results show high levels of both total phosphorus (TP) and total nitrogen (TN) between 1980 and 1990 for the upper four meters of the epilimnion. During this time TP ranged between 40 and 105 μg L^−1^ (mean = 73.4 μg L^−1^) and TN ranged between 800 to 1390 μg L^−1^ (mean = 1213 μg L^−1^). Levels of both TP and TN were high in the 1980s, related to sewage inputs that were subsequently controlled by the 1990s, leading to dramatic decreases in nutrients between 1990 and 2000 (TP range 22–54 μg L^−1^, mean 31 μg L^−1^ and TN range 520–1197 μg L^−1^, mean 818 μg L^−1^). This downward trend continued until 2005 when spikes in nutrients occurred as the result of a leak in a sewage pipe. Values between 2005 and 2013 had a TP mean of 34 μg L^−1^ while mean TN was 652 μg L^−1^. However, the highest concentrations between the time period 2005–2013 was 44 μg L^−1^ in 2006 for TP and 774 μg L^−1^ in 2009 for TN. Chlorophyll *a* measurements also showed a slow steady decline over time, and ranged between 45 μg L^−1^ (1989) and 11 μg L^−1^ (2004). Secchi depth had a range over time between 1.4 and 3.3 m. The deepest secchi depth was in 2009 at 3.3 m while the shallowest secchi depth was in 1989 with a depth of 1.4 m.

*Planktothrix* was primarily located in the epilimnion during the high nutrient period of the 1980s (Fig. 1). However, concurrent with the reduction of both TP and TN and a decrease in eutrophication and a general decrease in chlorophyll *a*, the light transmission increased as reported by a greater secchi depth measurement, and *Planktothrix* were reported occupying the metalimnetic zone. However, since the monitoring data [13] only captured the epilimnion, this is not easy to reconstruct. Microscopic measurements from epilimnetic phytoplankton samples showed that between 1984–1989 *Planktothrix* biovolume was, on average, 10,240 μg wet weight (WW) L^−1^ in the epilimnion while for the years 1990–2005 the average in the epilimnion decreased to 460 μg WW L^−1^. In 2005, concurrent with the sewage leak, *Planktothrix* populations increased to 5,236 μg WW L^−1^ in the epilimnion and remained high for 2 more years. In 2008–2010 *Planktothrix* disappeared only to return in 2011 with heavy blooms. Observation during sampling, however, was that the majority of the *Planktothrix* population was located in the metalimnion, depending on the depth of light penetration. Due to these reports, sampling for the pigment phycocyanin by NIVA has been instigated to better detect the presence of *Planktothrix* throughout the water column. Initial values in June of 2013 indicated low levels of phycocyanin (20 μm L^−1^) in the epilimnion while a peak of approximately 160 μm L^−1^ was found at ten meters depth. Historically, the presence of *Planktothrix* in the metalimnion rather than the epilimnion is a common occurrence and has often been cited both for Norway [15, 16] and worldwide [17, 18, 19].

### Sediment core

Dating of the core, as determined by the ^137^Cs peak representing the Chernobyl accident, resulted in a 0.7cm year^−1^ sediment accumulation rate. Sediment dry weight is on average 80% (SD = 2%) of wet weight and percent organic weight is on average 21% (SD = 2%).

### Primer and probe specificity

BLAST analysis for *Planktothrix* primers resulted in the identification of only matching *Planktothrix* strains. The results of qPCR amplification of *Planktothrix* chemotype standards for chemotypes 1, 5, 7 and 9 resulted in amplification only when using the appropriate matching primers, and were thereby considered to be specific. BLAST analysis for Chytrid-All primer set obtained 100% query cover for Chy-Lys2009 and Chy-Kol2008, the two chytrid sequences that the Chytrid-All primer had been designed to cover. The next closest results were sequences found in a terrestrial fungi associated with cotton plants with 96% coverage.

### Sediment DNA amplification and historical records

Extracted DNA from each centimeter of the sediment was analyzed for the four major chemotypes found in Norway. Chemotype 1 and chemotype 9 were amplified from the sediment DNA. In agreement with other research on the lake, the sediment DNA showed no amplification of chemotype 5 or chemotype 7 [12]. However, Sogge et al. [12] found an additional three isolates that were phylogenetically closely related with chemotype 9. qPCR testing of these three variants using the four chemotype primer sets resulted in amplification of only one culture, NIVA-CYA 597. This culture extract was amplified using chemotype 9 primers while none of the other chemotype primers resulted in amplification. No amplification was found for the other two *Planktothrix* cultures, NIVA-CYA 15 and 604, regardless of which primer sets were used. Therefore, when chemotype 9 is referred to, it is possible that this is actually a mix of chemotype 9 and a very closely related strain, NIVA-CYA 597, in unknown proportions. Although no other Kolbotnvannet cultures in NIVA-CCA proved to have novel chemotype sequences, it is possible that additional chemotypes have entered this lake since testing in 2013. It is known that *Planktothrix* are capable of horizontal gene transfer [20], which has the potential to increase diversity.

**Fig 2:**
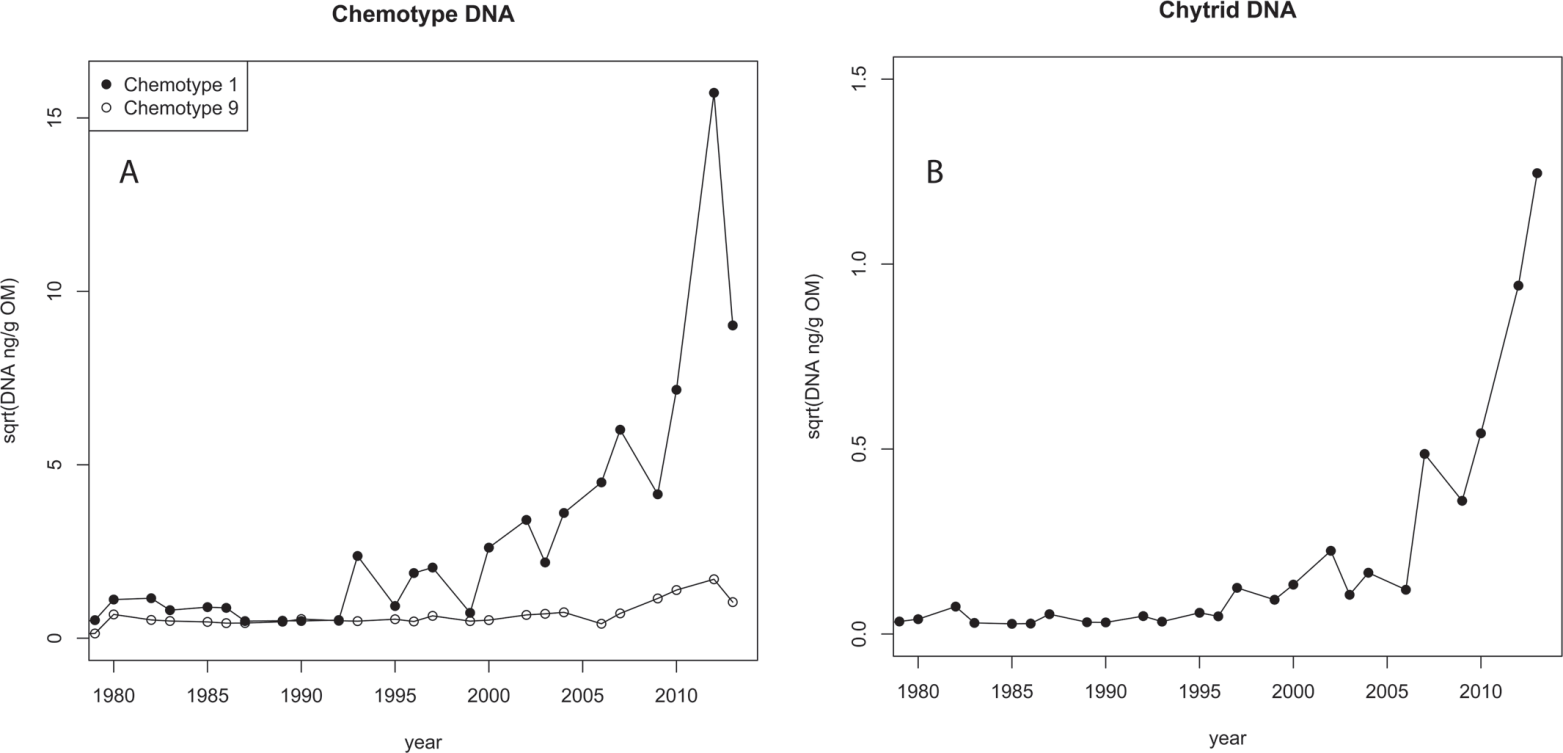
Chytrid and *Planktothrix* chemotype concentrations amplified from sediment of Kolbotnvannet, a lake in Southern Norway. DNA concentrations, based on organic matter (OM) content and square root corrected, for amplicons of *Planktothrix* and chytrids recovered from each centimeter of a sediment core from Kolbotnvannet dating from 1980 to 2013 for A) Chemotype 1 and Chemotype 9, and B) chytrids.

DNA concentrations were calculated based on the strain specific DNA standard curves and normalized to the organic matter (OM) fraction of each centimeter of the core. Concentrations varied between organisms and strains. Chemotype 1 concentrations ranging from 0.3 ng g OM^−1^ (1992) to 247.0 ng g OM^−1^ (2012) with an overall mean of 20.4 ng g OM^−1^ (SD = 51.0). Chemotype 9 concentrations had a range between 0.2 (2006) and 2.0 (2010) ng g OM^−1^ with a mean of 0.065 ng g OM^−1^ (SD = .02). Chytrid DNA ranged from 8.0 x 10^–4^ (1986) to 1.5 (2013) with a mean of 1.3 ng g OM^−1^ (SD = 0.4). Data was square root transformed to adequately handle non-detects, and to improve visual comparisons (Fig. 2). Overall chemotype 1 concentrations were low in 1979 and gradually increased over time. Chemotype 9 concentrations were consistently low and stable over the majority of the time course, with only a slight variation between 2006 and 2013. Chemotype 1 concentrations were much higher overall than chemotype 9, after 1990. Chytrid DNA concentrations were relatively low and stable until 2007 when they began a sharp increase.

**Fig 3:**
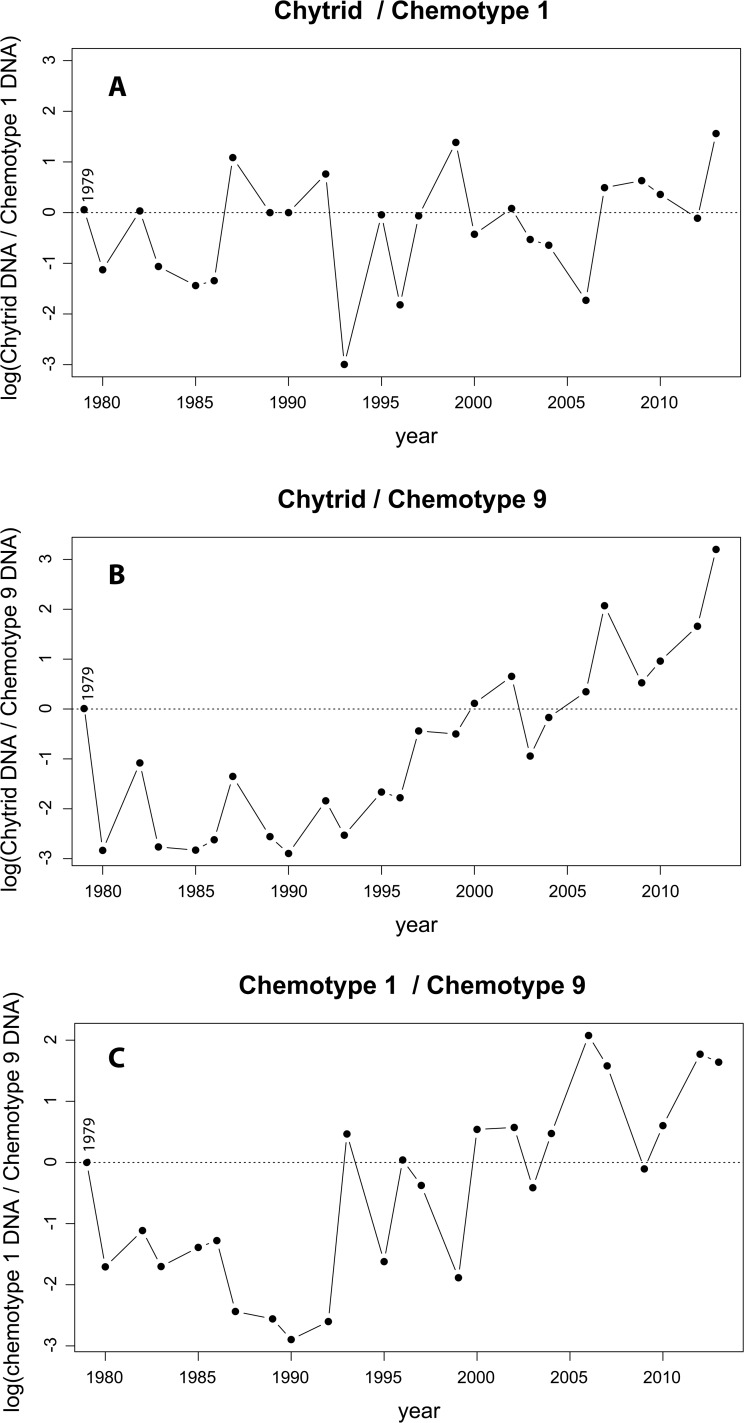
Comparison of chytrid parasite DNA and DNA from two *Planktothrix* chemotypes, 1 and 9. DNA ratios from chytrids and two chemotypes amplified from a lake sediment core. The ratios are normalized to those values representing 1979 (dotted horizontal line) and log converted values are compared. Relationships compared are A) chytrid and chemotype 1, B) chytrid and chemotype 9, and C) chemotype 1 to chemotype 9.

Comparison between chytrids and chemotypes (Fig. 3) are normalized to the 1979 level of chytrid to chemotype, creating a zero point in time to observe trends between chytrid and hosts or between hosts alone. After normalization, the data were log transformed to equalize positive and negative ratio effect sizes. The chytrid to chemotype 1 ratio (Fig. 3A) resulted in a dominant chemotype 1 signal (<1) in nine out of nineteen sampling points before 2005. However, following 2005, chytrid concentrations were greater (>1) than chemotype 1 in four out of five data points. Chytrid response to chemotype 9 (Fig. 3B) resulted in greater chemotype 9 (represented by the negative values <1) concentrations for fifteen out of nineteen sampling points before 2005 while after 2005 chytrid concentrations were always greater when compared with chemotype 9. A difference seen between the two chemotypes when tested against chytrids was that chemotype 1 had a higher variation overall. Comparison of the two chemotypes shows two distinct periods of time, early, from 1979 to 1992, and late from 1994 to 2013 (Fig. 3C). In the early period the ratio of chemotype 1 / chemotype 9 is <1, while the late period ratio >1.

Previously published data [10] showed that the four main chemotypes of *Planktothrix* in this region of Norway only have four oligopeptides in common, Anabaenopeptin B, Anabaenopeptin F, Desmethyl-microcystin LR, and Desmethyl-microcystin RR. Differences between the chemotypes are more common. While chemotype 1 has five unique oligopeptides, chemotype 5 has seven, chemotype 7 has only three, and chemotype 9 has six (Table 2 and S1 Dataset).

**Table 2:** Chemotype (Cht) properties.

Oligopeptide	Cht 1 n = 6	Cht 5 n = 10	Cht 7 n = 4	Cht 9 n = 25
Aeruginosin (559.5) -1				+
Aeruginosin (559.5) -2				+
Oscillaginin B (581.5)	+			
Aeruginosin (583.5) -1				+
Aeruginosin (583.5) -2				+
Aeruginosin (593.5)	+			
Oscillaginin A (615.5)	+			
Aeruginosin A (617.5)	+			
Anabaenopeptin C (809.6)		+	+	
Me-Anabaenopeptin C (823.6)		+	+	
Anabaenopeptin B (837.6)	+	+	+	+
Anabaenopeptin A (844.6)	+			+ (92%)
Anabaenopeptin F (851.6)	+	+	+	+
Oscillamide Y (858.6)	+			+ (92%)
Desmethyl-microcystin LR (981.6)	+	+	+ (75%)	+
Cyanopeptolin (1020.7)		+ (90%)		
Desmthyl-microcystin RR (1024.7)	+	+	+ (75%)	+ (96%)
Cyanopeptolin (1029.7)				+
Desmethyl-microcystin YR (1031.7)			+ (75%)	
Cyanopeptolin (1034.7)		+ (90%)		
Desmethyl-microcystin HtyrR (1045.6)		+		
Cyanopeptolin (1046.7)		+ (10%)		
Cyanopeptolin (1060.7)		+ (10%)		
Cyanopeptolin (1084.7)		+ (90%)		
Cyanopeptolin (1093.7)				+ (96%)
Cyanopeptolin (1098.7)		+ (90%)		
Cyanopeptolin (1109.6)				+ (4%)
Cyanopeptolin (1110.7)		+ (10%)		
Oscillapeptin G (1112.7)	+			
Cyanopeptolin (1124.7)		+ (10%)		
Cyanopeptolin (1126.7)			+ (25%)	
Cyanopeptolin (1142.7)			+	
Cyanopeptolin (1160.7)			+ (75%)	
Mean number of oligopeptides per chemotype	11	7 (sd 4)	3 (sd 1)	23 (sd 6)

Oligopeptides found in four *Planktothrix* chemotypes (1,5,7,9) from the Norwegian Institute for Water Research Culture Collection of Algae (n = strains tested). Number in parentheses indicates percentage of chemotype with that oligopeptide when it is different from 100%. Data used with permission from Rohrlack et al. [10].

For each isolate the identification number (ID) in the Norwegian Institute for Water Research Culture Collection of Algae, NIVA-CCA is given. The species names *P*. *rubescens* and *P*. *agardhii* are abbreviated with *P*. *rub*. And *P*. *aga*. respectively. Taken from Rohrlack T, Edvardsen B, Skulberg R, Halstvedt C, Utkilen H, et al. (2008) Oligopeptide chemotypes of the toxic freshwater cyanobacterium *Planktothrix* can form subpopulations with dissimilar ecological traits. Limnol. Oceanogr. 3(4): 1279–1293.

## Discussion

### *Planktothrix* chemotype occurrence

Of the major *Planktothrix* chemotypes, four (chemotypes 1, 5, 7, and 9) are typically found in this area of Norway while central European lakes support many more chemotypes [10, 21]. Our research found that only two out of the four common variants were present in the Kolbotnvannet sediment DNA tested. Sogge et al. [12] sequenced 82 strains that reside in the NIVA-CCA and have been isolated from lakes in this region of Norway. No *Planktothrix* filaments isolated from the Kolbotnvannet were found to be the variants chemotype 5 and chemotype 7. Phylogenetic analysis indicated that the cultures isolated from Kolbotnvannet were in agreement with the results of our sediment study where only chemotype 1 and chemotype 9 were well represented.

Sønstebø and Rohrlack [1] tested infection of 35 chemotype cultures of *Planktothrix* found in the NIVA culture collection using two chytrid cultures, NIVA-Chy-Lys2009 and NIVA-Chy-Kol2008, and found that all chemotypes were infected by chytrids with the exception of Cht7a, b, and c (see Fig. 2 in the Sønstebø and Rohrlack article). Because Cht7 was not amplified in this sediment, we assume chytrids were capable of infecting all Planktothrix chemotypes occurring in this lake. One important consideration in the use of historic sediment DNA is the potential for fragmentation and degradation. Typically, the best environmental conditions for DNA preservation are cold, anoxic sediments [22]. The conditions of sediment in Kolbotnvannet do not completely meet these conditions, however, DNA was recovered in our study for both *Planktothrix* and chytrids in fragments of at least 137 base pairs (bp) in length. Previous research [23] using cyanopeptoline *oci*B gene cluster, used in both studies, indicated that 161 bp fragments could be recovered from sediment in this region of Norway as far back as 80–150 years, depending on the sediment conditions. Therefore while it is possible that not all DNA deposited was recovered from the Kolbotnvannet sediment, comparing the recovered DNA from each chytrid and *Planktothrix* strain to each other reduces the direct effects of fragmentation.

During the early phase, between 1979 and 1990, Kolbotnvannet had high nutrient concentrations and eutrophic lake conditions (Fig. 1). During this time, the sediment data showed that two *Planktothrix* variants had a stable coexistence (Fig. 2A). Monitoring data indicates that this period of high nutrients gradually decreased and the lake entered a second period of moderate nutrient levels and deepening secchi depth between 1995 and 2013. During this later phase, the sediment data indicate a steady increase and dominance in chemotype 1 DNA concentrations, while at the same time chemotype 9 DNA remained low and stable. At no time did chemotype 5 or 7 appear. These results allow us to compare the sediment data with the laboratory results of De Bruin et al. [7] where parasitic pressure drives diversity.

### Chytrid—*Planktothrix* interactions

The findings of De Bruin et al. clearly showed no increase in chytrid fitness when presented with a diverse strain population of diatoms [7]. If we utilize changes in the accumulation of DNA (DNA concentrations) in the sediment layers to represent changes in fitness (the ability to survive and reproduce), our findings match those of De Bruin et al. only during our early phase. Two strains of *Planktothrix* coexisted (Fig. 2A) while chytrid concentrations remained stable (Fig. 2B).

As nutrients began to decline in the 1990s (late phase), *Planktothrix* chemotype 1 began to increase relative to chemotype 9 (Fig. 2A and Fig. 3C) while at the same time chytrids continued to remain stable relative to the chemotype. By 1995 chemotype 1 in Kolbotnvannet began a period of domination that lasted more than ten years. We hypothesized that if the chytrid—host interaction followed the hypothesis of De Bruin et al. [7] during the period where a single chemotype dominated in Kolbotnvannet, chytrid fitness should have increased rapidly after a short adaptation lag to completely remove chemotype 1, or to return to the conditions in the early phase. According to the work of De Bruin et al. [7], a dominant single genotype host would present the chytrids with a much smaller, simpler set of parameters, which would result in increasing parasite DNA concentrations and limiting the dominant host. Our results from the sediment DNA (Fig. 3A) did not follow this prediction, but to the contrary indicated that chemotype 1 was dominant over chemotype 9 in the presence of chytrids for an extended period of years (Fig. 3C). This period represents a much longer time frame than the 200 generations found by De Bruin et al. [7] to be required for the chytrid and single diatom shift. Due to the high growth rate ability of chytrids, the chytrids should have been able to overwhelm even a large chemotype 1 population and win the Red Queen arms race. However, this did not happen. Instead the relationship between chytrids and chemotype 1 showed stable coexistence (Fig. 3A). In the research of De Bruin et al. a single chytrid strain was used, which may not be the case in our environmental study. However, the laboratory study was designed to test how host populations responded to parasitic pressure in general in light of the Red Queen theory. The results were that host diversification protected host populations. They concluded that this would cause an arms race with diversification on both sides. The occurrence of two or more chytrid strains will not change the outcome. High parasitic pressure will continue to drive diversification in host population irrespective of whether this pressure is exerted by one or multiple chytrid strains. Quantifying all chytrids that infect *Planktothrix* is an estimate of this pressure. A decrease in host diversity at a constant parasitic pressure (as found in Kolbotnvannet) is either showing that the Red Queen theory is not applicable or that the host is increasingly better protected. Our findings indicate that the hypothesis of De Bruin et al. using the Red Queen theory is not upheld in our sediment study.

### Environmental stressors

Other factors might have played a role in maintaining chemotype1 dominance over other chemotypes and chytrids. The disease triangle presented by Gsell [2] describes the relationship not only as between parasite—host but also including environmental pressures. Gsell [2] describes the potential environmental stressors that affect both host and parasite but where adaptation to environmental stressors by either parasite or host can lead to increased relative fitness of one over the other. For example, low temperatures in the spring can be used as an advantage by diatoms enabling them a window of opportunity to bloom at a time when chytrids are limited by temperature constraints. The result is to release the diatoms from chytrid infection pressure [8]. Light is another environmental stressor for both chytrids and phytoplankton hosts. Bruning [24] found that there was a significant decrease in zoospore production on a light limited host. In the 1990s Kolbotnvannet *Planktothrix* changed habitat from the epilimnion to the metalimnion, at a time when nutrient and chlorophyll *a* concentrations in the lake had decreased allowing light to penetrate deeper into the water column. This shift downward by *Planktothrix* during periods of increasing light penetration is a common and a routinely found event around the world [15, 16, 17, 18, 19, 25] and not indicative of light limitation. *Planktothrix* in the metalimnion can take advantage of the environmental constraints of low light and lower temperatures on chytrid infection rates to increase growth. The ability to utilize low light levels could then be also viewed as a positive environmental adaptation by *Planktothrix* to avoid chytrid infection.

One noted difference between *Planktothrix* and *Asterionella* is the presence of intracellular oligopeptides found in *Planktothrix* [1]. These oligopeptides are responsible for internal defense against chytrid infection by inhibiting the chytrid proteases that are produced by the rhizoids as they extend through the cell [11]. They do not, however, protect the cell from cell death caused by chytrid parasitism. Instead they interfere with the life cycle or nutrient accumulation that would result in a decrease in production or maturation of zoospores and therefore a decrease in chytrid fitness. Rohrlack et al. [11] clearly showed in laboratory studies using wild type and mutant knock out strains of *Planktothrix* that the oligopeptides are a natural defense system used by the host against its chytrid predator. Table 2 presents the differences in cellular oligopeptides between the common Norwegian chemotypes 1, 5, 7 and 9. If chemotypes have adapted by increasing their internal defense system against parasitism then our study in Kolbotnvannet suggests that chemotype 1 shows a better defense against the chytrid infection than chemotype 9 in this lake. It might be considered that chemotype 5 and chemotype 7 were unable to present sufficient defense against resident parasites to support populations in this lake. It is possible that the internal oligopeptide diversity found in chemotype 1 could present chytrids with such a diverse internal environment that would challenge to chytrid adaption. Multiple oligopeptides in a cell could be considered similar to the multi-clonal population results found by De Bruin et al. [7]. While this study is unable to answer those questions, use of multiple oligopeptides for the protection of *Planktothrix* chemotypes against chytrid infection should be further researched.

## Conclusions

Our findings suggest that chytrid fitness was moderated during the early phase of our sediment data when the clonal differences of *Planktothrix* chemotypes controlled chytrid predation, which is in agreement with De Bruin et al. [7]. But this changed as nutrients declined in the later phase and one chemotype dominated while chytrids no longer drove diversity. One explanation is that *Planktothrix*, tolerant of low light and low temperatures, utilized the metalimnion where parasitic pressures were most likely released due to an increase in chytrid environmental stresses. In addition, the use of multiple internal oligopeptides by the host could have been effective defense against chytrid infection limiting chytrid growth during the period of dominance by chemotype 1. These oligopeptides might have evolved to present a similar defense as the clonal diversity has shown. Our sediment data results indicate *Planktothrix* was able to maintain a strong presence in Kolbotnvannet during the last 33 years, regardless of the presence of host specific parasitic chytrids. Therefore, while the early phase our study is in agreement with the findings of De Bruin et al. [7], the findings for the later phase of the chytrid—chemotype 1 relationship are counter to the findings of De Bruin et al. and to Red Queen theory. Other factors must play a role in the dominance of chemotype 1 during the later phase such as environmental mismatch between *Planktothrix* and chytrids, or the use of multiple oligopeptides in the host to defend against parasitism by chytrids.

This research is a unique opportunity to test the use of sediment DNA for a parasite—host analysis. While this study is limited to one Norwegian lake, it shows the possibility for using a time series from sediment DNA in ecological studies. This study is an important step in fully utilizing molecular techniques with sediment in paleoecology.

## Supporting Information

S1 Dataset Properties of clonal *Planktothrix* isolates. (DOC)
