# Supplementary material for: The Red Queen Race between Parasitic Chytrids and Their Host, Planktothrix: A Test Using a Time Series Reconstructed from Sediment DNA
Source: PLoS One. 2015 Mar 20;10(3):e0118738. doi: 10.1371/journal.pone.0118738 (PMC4368186; doi:10.1371/journal.pone.0118738)
Supplement: S1 Dataset — (DOC) [file pone.0118738.s001.doc]

S1_Dataset.doc
